# Artificial Liver Support System Improves Short- and Long-Term Outcomes of Patients With HBV-Associated Acute-on-Chronic Liver Failure: A Single-Center Experience

**DOI:** 10.1097/MD.0000000000000338

**Published:** 2014-12-02

**Authors:** Gang Qin, Jian-Guo Shao, Bin Wang, Yi Shen, Jian Zheng, Xian-Jin Liu, You-Yi Zhang, Yan-Mei Liu, Yan Qin, Lu-Jun Wang

**Affiliations:** From the Center for Liver Diseases (GQ, J-GS, BW, X-JL, Y-YZ, L-JW), Nantong Third People's Hospital, Nantong University; Department of Biostatistics (YS, Y-ML), School of Public Health, Nantong University, Jiangsu, China; Department of Paediatrics and Adolescent Medicine (JZ), Faculty of Medicine, The University of Hong Kong, Hong Kong; and Department of Internal Medicine (YQ), Singapore General Hospital, Singapore.

## Abstract

For patients with acute-on-chronic liver failure (ACLF), artificial liver support system (ALSS) may help prolong lifespan and function as a bridge to liver transplantation (LT), but data on its long-term benefit are lacking. We conducted this prospective, controlled study to determine the efficacy of ALSS and the predictors of mortality in patients with hepatitis B virus (HBV)-associated ACLF.

From January 2003 to December 2007, a total of 234 patients with HBV-associated ACLF not eligible for LT were enrolled in our study. They were allocated to receive either plasma exchange centered ALSS plus standard medical therapy (SMT) (ALSS group, n = 104) or SMT alone (control group, n = 130). All the patients were followed-up for at least 5 years, or until death.

At 90 days, the survival rate of ALSS group was higher than that of the control group (62/104 [60%] vs 61/130 [47%], respectively; *P* < 0.05). Median survival was 879 days in the ALSS group (43% survival at 5 years) and 649 days in the control group (31% survival at 5 years, log-rank *P* < 0.05). ALSS was found to be associated with favorable outcome of these patients by both univariate and multivariate analysis. Multivariate Cox regression analysis also revealed that lower serum sodium levels, higher grades of encephalopathy, presence of cirrhosis, hepatorenal syndrome, and higher model for end-stage liver disease scores were independent predictors for both 90-day and 5-year mortality due to ACLF.

Our findings suggest that ALSS is safe and may improve the short- and long-term prognosis of patients with HBV-associated ACLF.

## INTRODUCTION

Chronic hepatitis B (CHB) virus infection is a global public health concern. Acute-on-chronic liver failure (ACLF) in CHB is most commonly caused by acute severe exacerbation of CHB.^1^ In China, ACLF had been called severe chronic hepatitis until its diagnostic and treatment guideline was formally proposed by the Chinese Society of Hepatology in 2006.^2,3^ Other consensus definitions on ACLF were put forward by the Asian Pacific Association for the Study of the Liver (APASL) in 2009, American Association for the Study of Liver Disease (AASLD), and European Association for the Study of the Liver (EASL) in 2011.^4,5^

The prognosis of patients with ACLF is extremely poor with mortality rates ranging from 30% to 70% unless liver transplantation (LT) can be arranged on time. In the past 3 decades, a variety of artificial liver support systems (ALSSs), including plasma exchange (PE), molecular adsorbent recirculating system (MARS), and some other methods, has been employed to treat liver failure. Most research centers in China, including ours, started using the PE-centered ALSS nearly 2 decades ago. Meanwhile, MARS was widely applied for liver failure in western countries. Previous studies have demonstrated that the ALSS system might improve short-term survival in acute liver failure.^6^ There is, however, controversy on the effectiveness of ALSS for ACLF when LT is not available. Some studies, including a prospective controlled study, demonstrated that ALSS was safe, well tolerated, and may play roles in bridging to LT or recovery in well-defined patients with ACLF.^7–9^ It has been reported that the mean survival rates of patients with ACLF after 3 years were 33% in MARS-treated group and 15% in the control group.^10^ In contrast, other studies suggested that ALSS only provided a transient liver function support and the biochemical manifestation of ACLF may relapse and approach or even exceed the level before the previous ALSS treatment.^11^ Therefore, there is an urgent need to evaluate the long-term effect of ALSS on the patients with ACLF.

In this study, we undertook a prospective controlled study to test whether PE-centered ALSS treatment could improve short- and long-term prognosis of hepatitis B virus (HBV)-associated ACLF patients who were not eligible for LT, and identify predictive factors for the mortality of these patients.

## METHODS

### Study Design

An open-label, randomized, controlled parallel group design was conducted at the Center for Liver Diseases of Nantong Third People's Hospital, Nantong University. After qualifying for the trial, patients were randomly assigned to groups either given ALSS combined with SMT (ALSS group) or only SMT (control group). Randomization was performed by the Biostatistics Department of Nantong University based on the SAS module, using a ratio of 1:2 (later changed to 1:1) treatment:control in blocks of 6 patients. A set of blind envelopes were given to the center.

This study protocol conformed to the ethical guidelines of the 1975 Declaration of Helsinki and was duly approved by the ethics committee of Nantong Third People's Hospital, Nantong University. The potential benefits and risks of the use of ALSS and the nonavailability of the organ for LT were explained to the patients. Written informed consents for inclusion in the study were obtained from all patients (or in some instances, their closest relatives).

### Trial Entry

All patients presenting with HBV-associated ACLF (or HBV-associated severe chronic hepatitis before 2006) were screened. Patients were eligible for entry into the trial only when they met all of the following criteria: between 18 and 70 years of age; presumptive diagnosis of CHB, HBV-associated cirrhosis, or hepatitis B surface antigen (HBsAg) carrier; rapidly progressive hyperbilirubinemia with serum total bilirubin (TBIL) >10 mg/dL, within 28 days from symptom onset; coagulopathy with international normalized ratio (INR) >1.5 or plasma prothrombin activity <40%.^2,3^ The exclusion criteria were: acute HBV infection, superinfection with other viruses (hepatitis E, A, D, or C), superinfection with human immunodeficiency virus, and other causes of chronic liver failure such as alcohol- or drug-induced liver injury, severe gastrointestinal bleeding, coexistent hepatocellular carcinoma (HCC), or pregnancy.

### Baseline Assessment of Patients

The baseline data including age, sex, biochemical investigations, virological tests, abdominal ultrasound, and major complications were collected at the time of admission and summarized in Table 1. Severity of the liver disease was assessed by Child–Turcotte–Pugh (CTP) and model for end-stage liver disease (MELD) scoring systems.

**TABLE 1:**
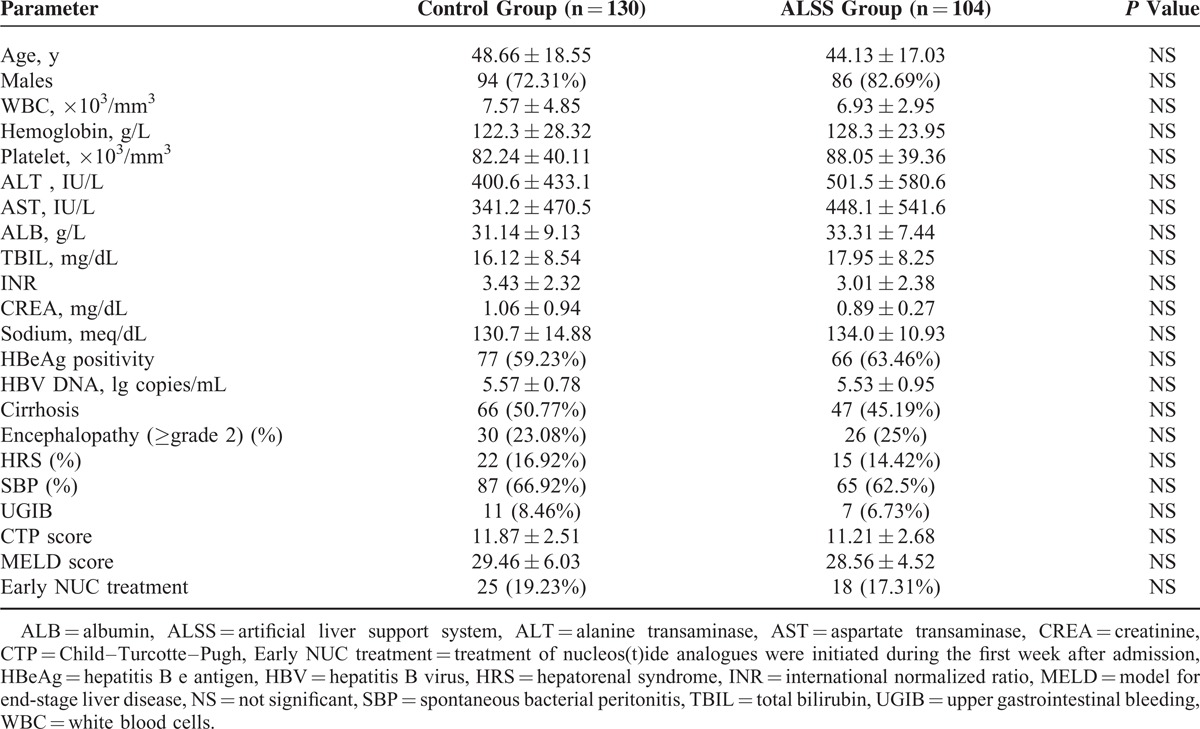
Demographic, Clinical, and Laboratory Variables of Included Patients at Admission

Serological tests for HBsAg and hepatitis B e antigen (HBeAg) were done by commercially available enzyme-linked immunoassays. The quantification of HBV DNA load was performed with the real-time polymerase chain reaction method (lower limit of detection 1000 copies/mL, Roche TaqMan assay).

Spontaneous bacterial peritonitis (SBP) and hepatorenal syndrome (HRS) was defined by International Ascites Club criteria.^12^ Hepatic encephalopathy (HE) with 4 grades (grade 1–4) was defined by the HE scoring algorithm (West Haven Criteria).^13^

### Description of Study Therapies

SMT was aimed to manage the precipitating events, support organ failure, and treat specific complications of ACLF. SBP was treated with antibiotics plus albumin infusion; HE was treated with oral nonabsorbable disaccharides such as lactulose;^14^ HRS was treated by using a combination of albumin infusion and administration of vasoactive drugs (mainly octreotide)^12^; upper gastrointestinal bleeding (UGIB) was treated with the combination of pharmacological and endoscopic therapy plus antibiotics. Proton pump inhibitors were administered when indicated. Other infections were assessed and treated with broad-spectrum antibiotics.

ALSS here was performed with plasma separator Plasmaflo KM-8800 (Kuraray, Tokyo, Japan) or Plasauto iQ-21 (Asahi, Tokyo, Japan). PE was conducted using the membrane separation method. The total volume of exchanged fresh plasma was around 3500 mL (40–60 mL/kg), using a 25–30 mL/min exchange rate.^15^ The ALSS sessions were scheduled as follows: 3 routine treatments were performed in the first 10 days after inclusion in the study (once per 3–4 days); extra treatments were offered according to the improvement of the patients. The methods of PE-centered ALSS were chosen based on individuals’ conditions. For patients with coagulopathy, PE was applied; for patients with encephalopathy, PE plus hemoperfusion or continuous hemodiafiltration was recommended; for patients complicated with HRS or imbalance of water or electrolytes, PE plus continuous hemodiafiltration were suggested.^9^ One hundred and four patients received 227 sessions (average 2 sessions/patient, ranging from 1 to 8 sessions) of ALSS treatment, with PE 197 times, PE plus hemoperfusion 21 times (for 11 patients), PE plus continuous hemodiafiltration 9 times (for 4 patients).

### Antiviral Therapy

Nucleos(t)ide analogues (NUCs) were prescribed according to individuals’ condition. During the first week after admission, 34 patients received 100 mg lamivudine (LAM) daily, 3 patients received 100 mg LAM plus 10 mg adefovir (ADV), 6 patients received 0.5 mg entecavir (ETV) daily (after ETV became available in China in 2006). Forty-three patients (18 in ALSS group and 25 in the control group) received early NUC treatment during the first week after admission.

During the follow-up from day 8 to 5 years, 90 patients (41 in ALSS group and 49 in the control group) with HBV DNA load over 1000 copies/mL were treated with NUC strategy, such as LAM, LAM plus ADV, or ETV. For those taking LAM as the initial antiviral treatment, most patients were required to receive ADV (plus LAM) or ETV (switch) regime. A few patients (9 in ALSS group and 12 in the control group) suspended NUC treatment due to lack of compliance. Ninety-five patients had continuous NUC treatment, which initiated at any time and sustained for at least one month to the end of follow-up or till death. The median durations of antiviral treatment were 26.5 versus 24 months in ALSS and control groups, respectively.

### Follow-Up

Clinical assessment and routine investigations were done daily during the first 15 days and then every 15 days till 90 days. The patients were followed-up at least twice a year after discharge. The primary endpoint of the study was 90-day survival and secondary endpoint was supposed to be 5-year or 10-year survival.

### Statistical Analysis

One retrospective cohort study from our center showed that the short-term (3 months) survival rates were 67% in ALSS group and 32% in the control group.^16^ Based on the assumption that the survival rates decreased approximately by half at 5 years, we calculated the sample size. Using Fisher exact test, taking α of 0.05 (2-sided) and power of 80%, the resulting sample size was 99 in each group.

Descriptive statistics are expressed as mean ± SD or number (%) unless otherwise stated. Comparison of continuous variables was done by student *t* test. For categorical variables, the χ^2^-square or Fisher exact test was used. Variables with a *P* value <0.05 at univariate analysis were included in the stepwise multivariate Cox regression analysis. Actuarial probability of survival was calculated by Kaplan–Meier graph and compared by log-rank test. Analysis was done according to intention-to-treat.

Statistical significance of all tests was defined as *P* < 0.05 by 2-tailed tests. All analyses were performed using the STATA statistical software (version 12.0; StataCorp, TX).

## RESULTS

### Study Patients

**FIGURE 1:**
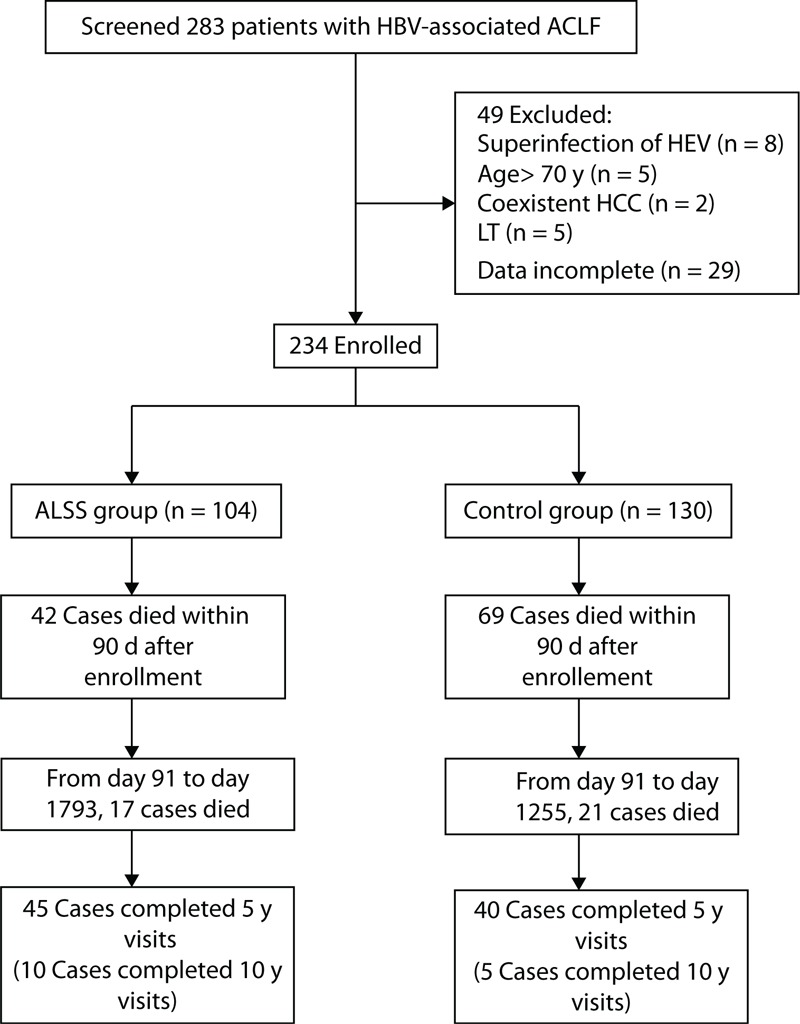
Study profile. HCC = hepatocellular carcinoma, LT = liver transplantation.

From January 2003 through December 2007, 283 patients presenting with HBV-associated ACLF were screened. After baseline investigations, 234 patients were enrolled and randomized; 130 (56%) assigned to the control group and 104 (44%) to ALSS group (Figure 1). At the beginning, the assignment ratio was 2:1 with more patients assigned to the control group. A year later, the ratio was changed to 1:1 in order to recruit more patients to the ALSS group after our preliminary data analysis suggested some benefits for ALSS.

Liver transplantation was offered to 2 patients in the control group shortly after enrollment, whose data were still analyzed according to originally randomized treatment assignment.

All the patients in the study were followed-up for 5 to 10 years, or until death (Figure 1). No patients were lost to follow-up in this study.

### Baseline Characteristics

The median duration of hospital stay was 25 (range 6–177) days. Baseline characteristics such age, sex, HBV DNA level, TBIL, INR, presence of cirrhosis and complications, CTP and MELD scores, and early antiviral treatment with NUCs in the 2 patient groups were similar (Table 1).

### Effects of ALSS on Survival

**FIGURE 2:**
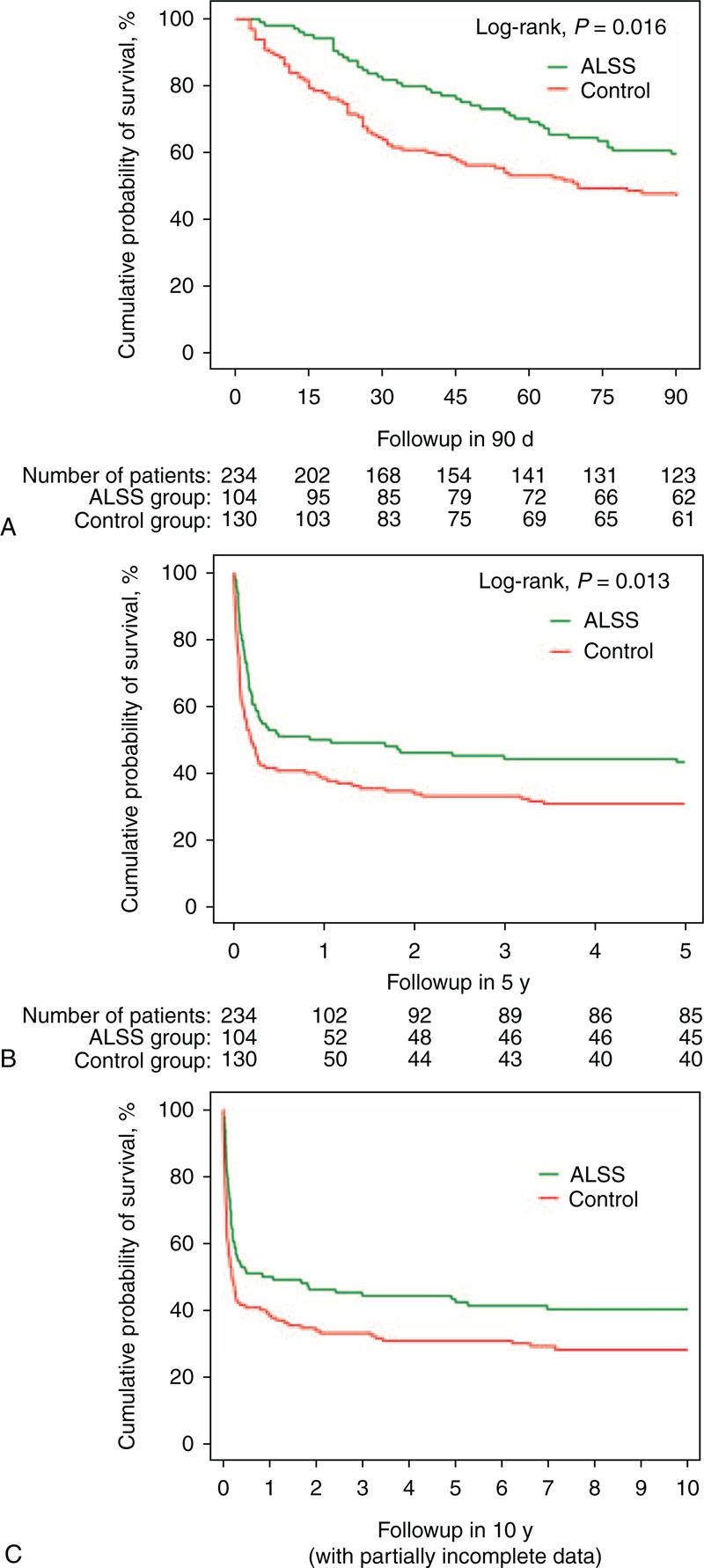
Cumulative survival in ACLF patients treated with SMT plus ALSS compared with SMT alone (control) over follow-ups of (A) 90 days, (B) 5 years and, (C) 10 years. ACLF = acute-on-chronic liver failure, ALSS = artificial liver support system, SMT = standard medical therapy.

Figure 2A shows that the survival rates after 90 days were 60% (62/104) in ALSS-treated patients and 47% (61/130) in the control group. The 5-year cumulative survival rates of the ALSS and control groups were 43% (45/104) and 31% (40/130), respectively. The cumulative survival probability was significantly higher in the ALSS group at both evaluation endpoints (log-rank *P* < 0.05, Figure 2A and B).

In our study, 149 (64%) patients died during the follow-up of 5 years, whereas most of the deaths (75%, 111/149) occurred during the first 90 days. Complications of progressive liver failure included HRS (16%, 37/234), HE equal or greater than grade 2 (25%, 56/234), UGIB (8%, 18/234), and SBP (65%, 152/234). The deaths resulted from one or more of the complications in the first 90 days. From day 91 to 5 years, 31% (38/123) of the remaining patients succumbed. Twenty-seven patients died from the complications of liver cirrhosis (infection, bleeding, encephalopathy, or HRS), whereas 11 patients died from HCC.

The median survival was 879 days in the ALSS group (43% survival after 5 years) and 649 days in the control group (31% after 5 years, log-rank *P* = 0.02). ALSS-treated patients gained 0.63 (95% CI: 0.04 to 1.22) life years, determined by the bootstrap method.

The incomplete 10-year follow-up data suggested that the difference of survival rates between the ALSS group and the control group remained stable over time (Figure 2C).

### Predictors of Short-Term Mortality

Table 2 shows the unified relationship of baseline factors with 90-day postadmission mortality due to ACLF. The elder ages, lower levels of platelets, hemoglobin, sodium or albumin, higher levels of white blood cells, TBIL, INR or creatinine, and the presence of cirrhosis, encephalopathy (≥grade 2), HRS, or SBP revealed individual associations with short-term mortality due to ACLF (*P* < 0.05). Intriguingly, lack of ALSS treatment was found to be associated with the unfavorable outcome of the patients here (*P* < 0.05).

**TABLE 2:**
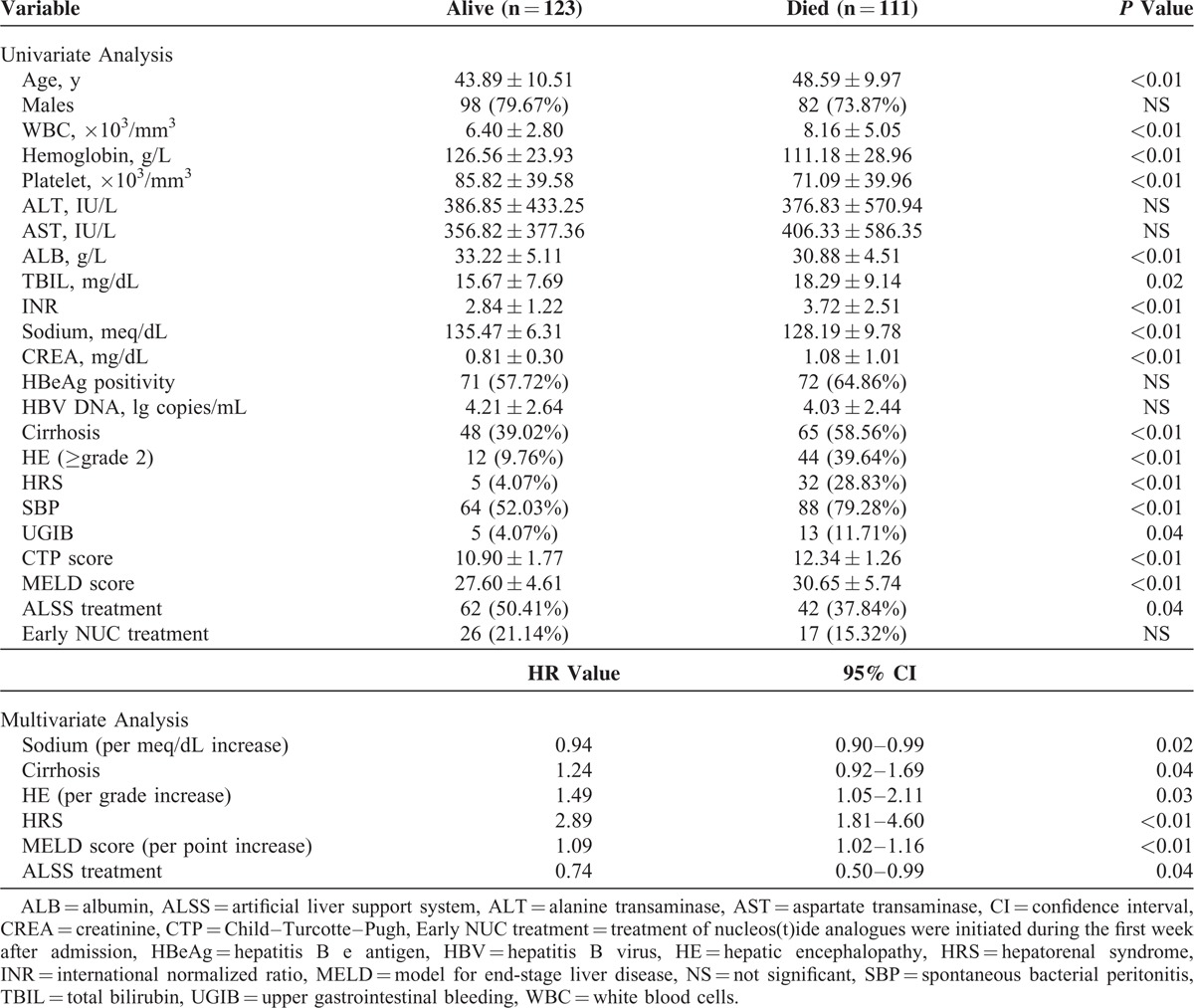
Univariate and Multivariate Analysis of Baseline Predictors of 90-Day Mortality

When the above significant variables were entered into multivariate stepwise Cox regression analysis, only the lower serum sodium levels (HR 0.94; 95% CI 0.90–0.99), higher grades of encephalopathy (HR 1.49; 95% CI 1.05–2.11), presence of cirrhosis (HR 1.24; 95% CI 0.92–1.69), HRS (HR 2.89; 95% CI 1.81–4.60), higher MELD scores (HR 1.07; CI 1.02–1.16), and lack of ALSS (HR 0.74; CI 0.50–0.99) were identified as independent predictors for 90-day mortality due to ACLF (*P* < 0.05) (Table 2).

### Predictors of Long-Term Mortality

On univariate analysis, the elder ages; lower levels of platelets, hemoglobin, sodium, or albumin; higher levels of white blood cells;TBIL, INR, or creatinine; presence of cirrhosis, encephalopathy (≥grade 2), HRS, or SBP; and lack of ALSS treatment were also found to be associated with fatal long-term outcome (*P* < 0.05). Continuous antiviral treatment with NUCs was found to be associated with the favorable outcome of the patients (*P* < 0.05).

After multivariate adjustment, the elder ages (HR 1.07; 95% CI 1.03–1.11), lower serum sodium levels (HR 0.93; 95% CI 0.87–0.99), higher grades of encephalopathy (HR 2.29; 95% CI 1.31–3.99), presence of cirrhosis (HR 1.65; 95% CI 0.98–2.77), HRS (HR 3.40; 95% CI 2.09–5.53), higher MELD scores (HR 1.07; CI 1.02–1.16), and the lack of ALSS (HR 0.67; 95% CI 0.48–0.94) or continuous NUC treatments (HR 0.74; 95% CI 0.49–1.10) were identified as independent predictors for 5-year mortality after ACLF attack (*P* < 0.05) (Table 3).

**TABLE 3:**
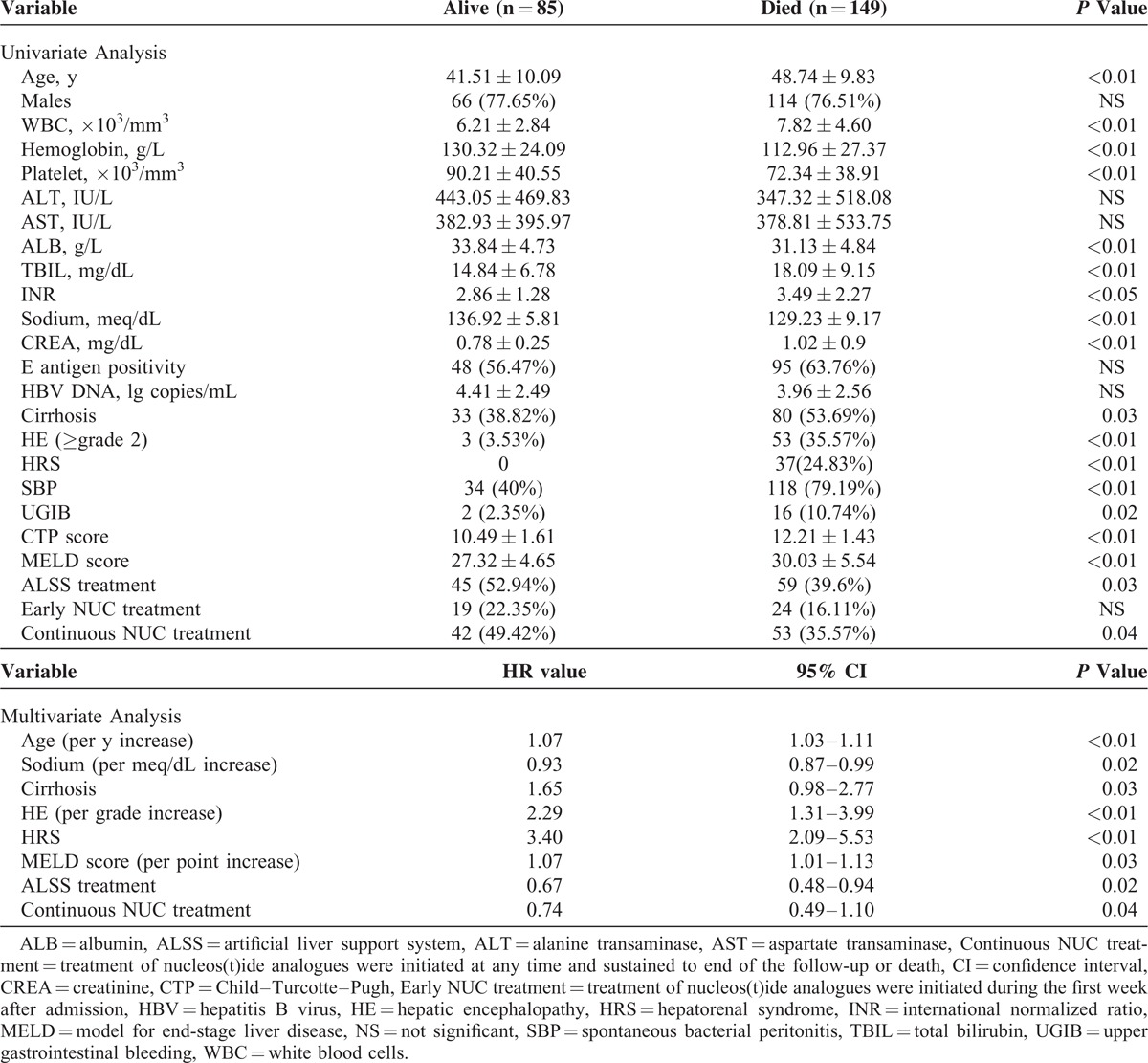
Univariate and Multivariate Analysis of Baseline Predictors of 5-Year Mortality

### Adverse Events

Common complications that occurred during ALSS therapy included skin rash (26.92%, 28/104), hypotension (20.19%, 21/104), and blood coagulation in perfusion apparatus (10.58%, 11/104). All patients tolerated therapy with parameter modification or early discontinuation. There were no differences in the number of patients who developed the complications that could potentially arise from the application of the ALSS therapy (Table 4).

**TABLE 4:**
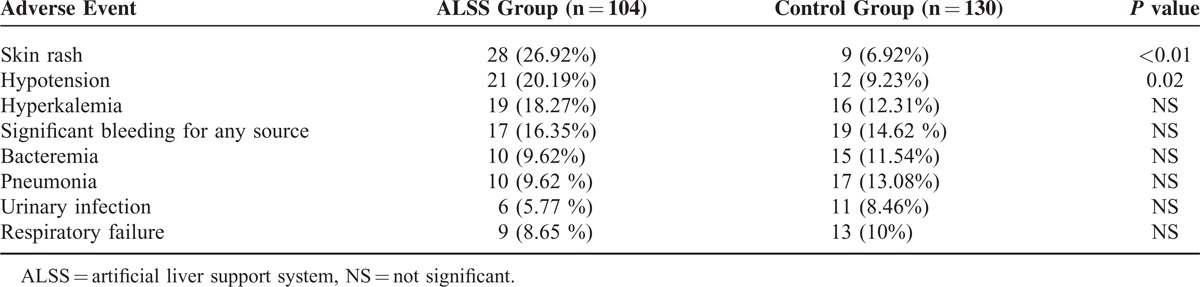
Adverse Events During the 90-Day Study Period

## DISCUSSION

Liver transplantation is the best procedure to treat ACLF, with a perioperative mortality rate of <3% and 1-year survival rate of exceeding 80% for recipients in some major transplant centers.^17^ Recent studies have suggested that ALSS (PE or MARS) could provide extra time for the patients with ACLF as a bridge to LT.^9^

Our hospital is a local teaching hospital that services a population of 7,000,000 in an area of high HBV endemicity.^18^ Since we started performing LT in our center in 2002, ACLF has been one of our main indications of LT. However, as a result of limited number of donor livers, our surgeons performed only 7 cases (including 2 cases in this study) of HBV-associated ACLF and 24 cases of other liver diseases in the period from January 2003 to December 2007. The increasing discrepancy between the number of potential candidates for LT and donor liver availability suggests that some therapeutic alternatives for these patients should be necessary.

PE-centered ALSS is an attractive approach for the treatment of ACLF. First, it has been shown to remove overabundant toxic substances and correct the severe coagulopathy.^19^ Second, there are quite a few studies showing that it improves HE in patients with ACLF.^20,21^ Third, there are several studies suggesting a potential beneficial effect of PE-centered ALSS on survival in patients with ACLF.^19,22^ We report here the largest prospective controlled study using PE-centered ALSS in patients with ACLF. We found that the ALSS therapy sustained the patients’ lives with a mean time of more than half a year. There is a clear relationship between ALSS therapy and both short- and long-term survival benefits for patients with HBV-associated ACLF. Interestingly, although most deaths of ACLF patients occurred in the first 90 days, our results showed that the 5-year living opportunity of 69% for the patients who survived the first 90 days was promising. Salvaging ALSS here seemed to be carried out timely to support liver function and win better survival opportunity for these patients.

It is important to point out that our definition of ACLF is subtly different from the criteria proposed by AASLD, EASL, or APASL. The current working definition as proposed by a working group from AASLD and EASL emphasized the occurrence of “cirrhosis” and “multisystem organ failure.”^5^ APASL recommended jaundice with serum bilirubin level >5 mg/dL as 1/2 mandatory criteria for diagnosis of ACLF.^4^ For our definition of ACLF, the cut-off level of serum bilirubin was 10 mg/dL, and cirrhosis and multiorgan failure were not taken as mandatory criteria, according to the Chinese guidelines.^2,3^ The latter explained why the serum creatinine and albumin were better preserved and the survival rate was higher in our patients. In addition, all cases described here were ACLF based on chronic HBV infection, whereas 70% of the cases in the study by Hessel et al^10^ were on alcoholic liver disease. The etiology of ACLF might also contribute to our survival result different from other reports.

Infection plays an important role in the pathogenesis and mortality in ACLF.^23^ One recent study showed that independent predictors of poor 30-day survival of ACLF included infection-related ACLF (I-ACLF), admission values of high MELD, and low albumin.^24^ In our study, the incidence of SBP was quite high in the patients. But SBP was not identified as an independent predictor of mortality by multivariate analysis. Explanation might be that the sample size was not big enough and the infection here was not as severe as in I-ACLF, which was defined by the presence of 2 or more organ failures.

HBV replication is one of the key factors causing severe liver damage. Early antiviral treatment with nucleos(t)ide analogues may improve the short- and long-term prognosis of patients with HBV-associated ACLF.^25^ It has been well accepted that early and rapid reduction in HBV DNA may be the essence of therapy for HBV-associated ACLF.^26^ However, some factors, including the limited options (only LAM at the beginning, then ADV and ETV available in China from 2005 and 2006, respectively), the need for long-term (perhaps indefinite) treatment, the risk of viral resistance, the unknown long-term safety (eg, renal safety), and high costs restricted the use of NUCs in our study.^22,27,28^ Only up to 20% of patients received early NUC treatment. The small proportion might partially explain why the early NUC treatment was not identified as a prognostic indicator for short-term outcome. However, with the increased number of patients who received antiviral treatment during the follow-up, the continuous NUC treatment was found to be an independent prognostic factor for long-term survival.

There has been concern about the safety and applicability of the extracorporeal therapy in critically ill patients. Our results demonstrated that the use of the ALSS in this clinical context was not associated with an increase in severe adverse events.

Our study has some limitations. First, our clinical research was not registered as a clinical trial in a public registry. It was partially because the Chinese Food and Drug Administration had not been founded until 2003. Second, we changed allocation ratio during the study. The unequal number of cases in the 2 groups may add complexity when compared with using balanced allocation. But it was also used elsewhere with acceptable reasons.^29^ Another limitation is that the antiviral therapy in this study varied widely in the patients, in terms of initiation time, drug selection, treatment duration, and regime changes.

## CONCLUSION

In summary, our study, for the first time, has clearly demonstrated the efficacy and safety of PE-centered ALSS in supporting liver function and extending the living time for these patients. The indications for ALSS should be broadened for HBV-associated ACLF patients concerning the long-term benefits without LT. In this regard, we believe that more patients with HBV-associated ACLF will achieve a prolonged life if salvaging ALSS is applied.
